# What benefit can be obtained from magnetic resonance imaging diagnosis with artificial intelligence in prostate cancer compared with clinical assessments?

**DOI:** 10.1186/s40779-023-00464-w

**Published:** 2023-06-26

**Authors:** Li-Tao Zhao, Zhen-Yu Liu, Wan-Fang Xie, Li-Zhi Shao, Jian Lu, Jie Tian, Jian-Gang Liu

**Affiliations:** 1 School of Engineering Medicine, Beihang University, Beijing, 100191 China; 2 School of Biological Science and Medical Engineering, Beihang University, Beijing, 100191 China; 3 CAS Key Laboratory of Molecular Imaging, Institute of Automation, Beijing, 100190 China; 4 University of Chinese Academy of Sciences, Beijing, 100080 China; 5 Department of Urology, Peking University Third Hospital, Peking University, 100191 Beijing, China; 6 Key Laboratory of Big Data-Based Precision Medicine (Beihang University), Ministry of Industry and Information Technology of the People’s Republic of China, 100191 Beijing, China; 7 Beijing Engineering Research Center of Cardiovascular Wisdom Diagnosis and Treatment, Beijing, 100029 China

**Keywords:** Clinically significant prostate cancer, Adverse pathology, Radiomics quality score, Artificial intelligence, Magnetic resonance imaging

## Abstract

The present study aimed to explore the potential of artificial intelligence (AI) methodology based on magnetic resonance (MR) images to aid in the management of prostate cancer (PCa). To this end, we reviewed and summarized the studies comparing the diagnostic and predictive performance for PCa between AI and common clinical assessment methods based on MR images and/or clinical characteristics, thereby investigating whether AI methods are generally superior to common clinical assessment methods for the diagnosis and prediction fields of PCa. First, we found that, in the included studies of the present study, AI methods were generally equal to or better than the clinical assessment methods for the risk assessment of PCa, such as risk stratification of prostate lesions and the prediction of therapeutic outcomes or PCa progression. In particular, for the diagnosis of clinically significant PCa, the AI methods achieved a higher summary receiver operator characteristic curve (SROC-AUC) than that of the clinical assessment methods (0.87 vs. 0.82). For the prediction of adverse pathology, the AI methods also achieved a higher SROC-AUC than that of the clinical assessment methods (0.86 vs. 0.75). Second, as revealed by the radiomics quality score (RQS), the studies included in the present study presented a relatively high total average RQS of 15.2 (11.0–20.0). Further, the scores of the individual RQS elements implied that the AI models in these studies were constructed with relatively perfect and standard radiomics processes, but the exact generalizability and clinical practicality of the AI models should be further validated using higher levels of evidence, such as prospective studies and open-testing datasets.

## Background

Prostate cancer (PCa) is one of the most prevalent cancers among men, especially in the United States, with the highest incidence and second highest mortality rate [1–3]. In China, PCa has three epidemiological characteristics. First, it ranks highest in the annual increase in both morbidity and mortality in men [4]. Second, the ratio of mortality to morbidity of PCa is higher than that in some Western countries [2–4]. Third, the proportion of patients with high-risk advanced PCa is high due to limited prostate specific antigen (PSA) screening [1, 5]. Two challenges in PCa diagnosis and treatment are the precise diagnosis of PCa and the prediction of the therapeutic outcomes or PCa progression, which have attracted extensive interest from researchers [6–12].

Invasive biopsy is a common method used in clinical practice to monitor PCa [13–16]. However, the randomness of the needle position for biopsy sampling limits the ability of the biopsy to capture the spatial state of the lesions and therefore, leads to the omission of true tumors. Additionally, patients who undergo biopsies may have some reactions, such as bleeding, pain, infection, and even life-threatening sepsis in severe cases [16, 17]. On the other hand, medical imaging can provide a comprehensive macroscopic description of the tumor phenotype and peritumoral context, which can be a compensatory and noninvasive approach to provide information by quantifying tumor progression before, during, and after treatment [18, 19]. Therefore, characterization based on medical imaging is a practical method for quantifying the heterogeneity of PCa and potentially facilitating the development of precision medicine.

Magnetic resonance imaging (MRI) is a common medical imaging methodology with high spatial resolution and can also describe different physiological and anatomical characteristics based on various sequences. For example, T2-weighted imaging (T2WI) can describe the anatomical structures of tumors and is, therefore, useful for delineating the profiles and appearances of suspicious lesions. Diffusion-weighted imaging (DWI) and apparent diffusion coefficient (ADC) maps derived from DWI can reflect the degree of random motion of water molecules related to the tumor’s aggressiveness. Additionally, the dynamic contrast-enhanced sequence can be used for the functional and physiological assessment of tumors with the guide of a gadolinium contrast agent. Further, compared with computed tomography (CT) and positron emission tomography (PET), MRI has no radiation risk and has thus been widely used for tumor diagnosis and monitoring [15, 18, 20–24].

In clinical diagnosis, magnetic resonance (MR) images of prostate lesions are visually assessed according to the prostate imaging reporting and data system (PI-RADS) guidelines based on some visually quantitative features of lesions (e.g., location, shape, size, and intensity) [25, 26]. The visual assessment of MR images plays an important role in the diagnosis but has several limitations [6, 27–30]. First, the visual assessment of MR images is greatly dependent on the high-level expertise of radiologists, leading to discrepancies in the assessment results. Second, some features of MR images reflecting tumor heterogeneities cannot be observed on the visual assessment. Third, the visual assessment is qualitative or semi-quantitative [26]. These limitations may lead to a decrease in the accuracy and robustness of PCa diagnoses.

Artificial intelligence (AI) is a data-driven method. In other words, after being trained with many samples, an AI model can automatically select the optimal feature pattern to accurately predict the novel samples. Thus, when AI methodology is used to analyze medical images of PCa, it can mine fine and deep information that may reflect relatively complete heterogeneities of the suspicious lesions, regardless of whether this information is visually representable. Due to its advantages in analyzing medical images, AI methodology has been widely applied to aid in the diagnosis and treatment of PCa [12, 31–35] and other malignancies [13, 36–46]. Increasing evidence supports the ability of AI methods to facilitate precise diagnosis and treatment of tumors. In fact, some AI software that can help identify PCa has been approved by the Food and Drug Administration (FDA). For example, ProstatlD software aims to interpret prostate MRI and assist radiologists in identifying suspicious PCa regions and analysing their likelihood of malignancy [47]. AI-Rad Companion Prostate MR software aims to assist the radiologists in automatically segmenting prostate, estimating volume and manually delineating the location of lesions with MR images, which can be used to support the planning of biopsies [48]. Additionally, one AI software, i.e., Paige Prostate software, is developed with pathological images instead of MRI. This software is designed to aid pathologists in detecting suspicious areas on prostate biopsy images and further assessing the likelihood of malignancies [49].

The National Comprehensive Cancer Network (NCCN) reported that MRI could generally guide PCa monitoring [15]. In clinical practice, patients with PI-RADS scores 3−5 are recommended to undergo biopsies for further pathological confirmation [17]. However, patients with a PI-RADS score 3 are equivocal in detecting clinically significant prostate cancer (csPCa). As a result, it may lead to low specificity and, therefore, overdiagnosis [6, 28, 30]. Additionally, according to the NCCN, some patients are recommended to undergo radical prostatectomy (RP) or other therapies. However, some of these patients may have high risks of the presence of adverse pathology (AP), disease recurrence, and subsequent metastasis [10, 50–53]. Advance identification of these patients before treatment may be beneficial to their prognoses. To address these problems, many studies have constructed a variety of AI models for the diagnoses and treatments of PCa, such as the diagnosis of csPCa [54, 55], prediction of Gleason grade [56], prediction of biochemical recurrence (BCR) [57], and extracapsular extension (ECE) [58]. They have compared the performances of these models with those of visual assessments based on PI-RADS or other clinical assessments. Currently, most of the published reviews focus on the analysis of the modeling processes and tasks of AI methods [59–61]. However, reviews comparing the performance between AI and clinical assessment methods are limited, though they can highlight the clinical value and potential of AI methodology to aid clinicians in precisely diagnosing PCa and predicting therapeutic outcomes or progression of PCa.

To bridge this gap, in the present study, we focused on studies that reported results from both AI and clinical assessment methods. Then, we analyzed and summarized these studies, comparing the diagnostic and predictive performance for PCa between AI and common clinical assessment methods based on MR images and/or clinical characteristics, thereby exploring the potential of AI in the diagnosis and treatment of PCa. Specifically, we compared the performance between AI and clinical assessment methods for the diagnosis and prediction fields of PCa. In particular, we quantitatively compared the abilities of these two methodologies to diagnose csPCa and predict AP. Additionally, the quality of the studies was assessed based on the radiomics quality score (RQS).

## AI pipeline on the diagnosis and prediction fields of PCa

This study focused on two fields of AI application to PCa: the diagnosis field, which refers to the identification of malignant lesions and stratification of PCa risk; and the prediction field, which refers to the prediction of therapeutic outcomes or progression of PCa.

**Fig. 1:**
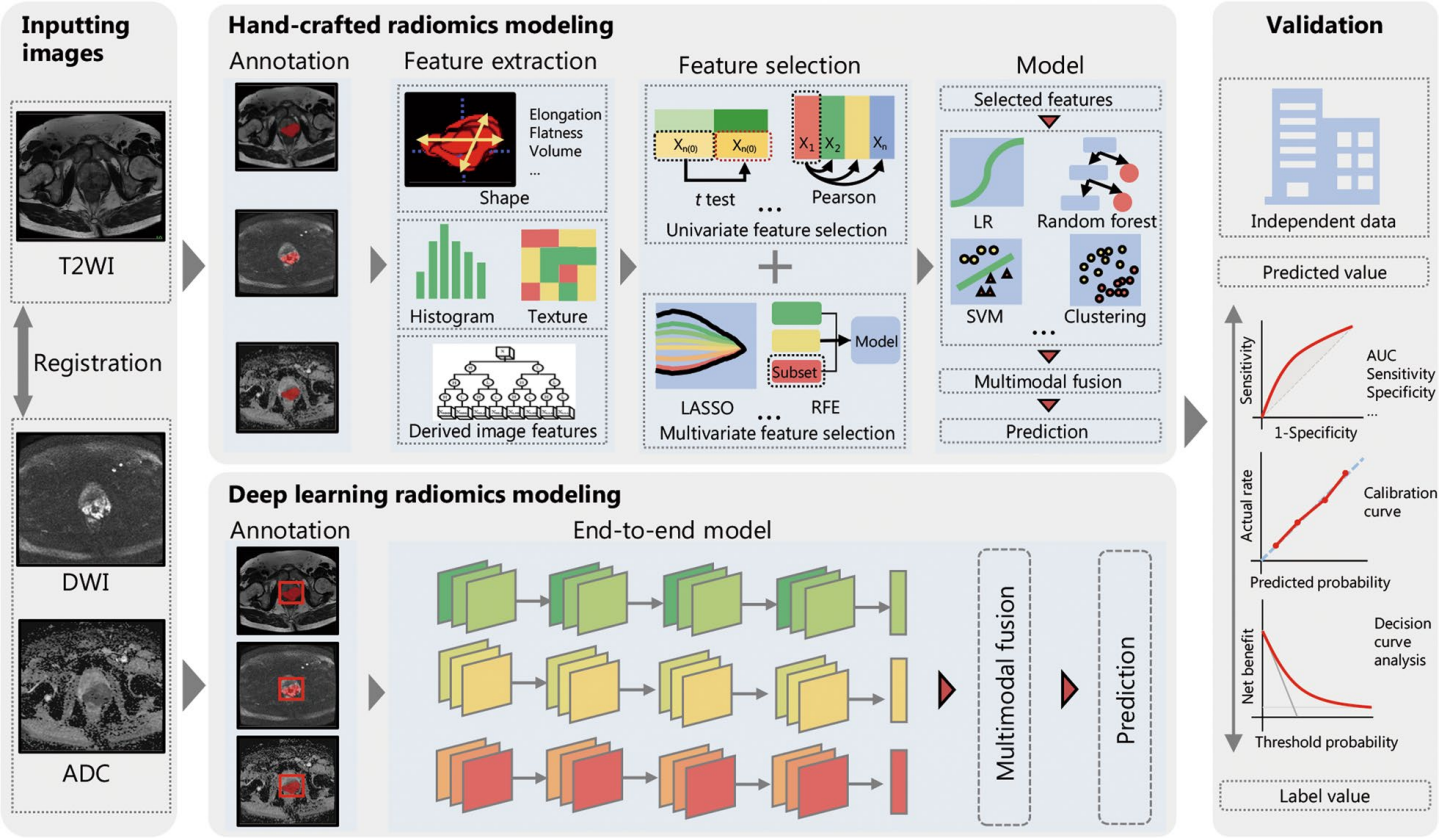
Pipeline of the classification process of artificial intelligence. There are three discrete steps in the pipeline: inputting images (i.e., image acquisition and pre-processing), model development, and model performance validation. Generally, there are two main approaches to developing AI models: the hand-crafted radiomics method and the deep learning radiomics method. For the hand-crafted radiomics model, there are four steps: annotation, feature extraction, feature selection and modeling with traditional machine learning methods. For the deep learning radiomics model, the images can be fed into the end-to-end model to output the risk probability of adverse outcomes. ADC apparent diffusion coefficient, AUC area under the receiver operating characteristic curve, DWI diffusion-weighted imaging, LASSO least absolute shrinkage and selection operator, LR logistic regression, RFE recursive feature elimination, T2WI T2-weighted imaging, SVM support vector machine

The pipeline of the process of the AI methodology includes several discrete steps: image acquisition and pre-processing, model development, and model performance validation. Generally, there are two main approaches to developing AI models for medical imaging analysis: the hand-crafted radiomics method and the deep learning radiomics method (Fig. 1).

The hand-crafted radiomics method can provide a set of high-throughput features. Specifically, prostate lesions are first manually delineated in MR images. Then the features including shape, histogram, and textural features are extracted from the delineated lesions in the original MR images and their derived images (e.g., wavelet). The shape and histogram features refer to the metrics characterizing the shape (e.g., size, volume and flatness) and histogram (e.g., mean, entropy, and skewness), respectively. The textural features were extracted by the calculation matrixes reflecting the distribution of gray intensity, such as Gray Level Co-occurrence Matrix, Gray Level Size Zone Matrix, and Gray Level Run Length Matrix. These extracted features are fed into traditional machine learning models such as logistic regression, support vector machine (SVM), and random forests (RF) after a feature selection step, which finally outputs a quantitative score with a value ranging from zero to one, indicating the risk probability of adverse outcomes such as csPCa and BCR. Compared with the deep learning radiomics method, the hand-crafted radiomics method is simple owing to fewer parameters in traditional machine learning models and is, therefore, easier to achieve. Additionally, specific features have relatively evident semantic information, thereby increasing the interpretability of the models [39]. However, it requires precise manual delineation of the tumor slice by slice, which is time-consuming, laborious, and can lead to subjective disagreements among different radiologists.

In contrast, the deep learning radiomics method can automatically extract the features of medical images without requiring the precisely manual delineation of lesions. Specifically, deep learning radiomics models have been constructed using various networks, such as ResNet [62], MobileNet [63], and ShuffleNet [64]. Deep learning radiomics models use original images or rectangular volumes of interest containing lesions as inputs, from which image features can be extracted directly through convolution operation of networks. Therefore, precise manual segmentation of lesions can be avoided. Similar to the hand-crafted radiomics method, the deep learning radiomics models output a value indicating the risk probability of adverse outcomes. Thus, the deep learning radiomics method is particularly suitable for the analysis of a large number of samples. Additionally, owing to multi-layer construction, deep learning radiomics models can mine deep and subtle image information that can accurately characterize the heterogeneity of PCa, thereby showing excellent performance for predicting adverse outcomes. Both hand-crafted and deep learning radiomics methods can be used for the diagnosis and prediction fields of PCa. They can be employed alone or in combination via the fusion of features or integration of models. In this study, AI models constructed based on the hand-crafted and deep learning radiomics methods are referred to as hand-crafted (HC) and deep learning (DL) models, respectively.

## Comparing the performances of AI and clinical assessment methods in the diagnosis field of PCa

The application of AI in diagnosis fields of PCa based on MR images has attracted extensive interest. In clinical practice, diagnostic tasks for PCa mainly include the risk stratification of prostate lesions, such as PCa detection (i.e., the discrimination between benign and malignant lesions) and csPCa detection (i.e., the discrimination between non-csPCa and csPCa). According to the International Society of Urological Pathology Gleason grade group (GGG), patients with GGG < 1 and GGG ≥ 1 were defined as having benign and malignant lesions, respectively. Patients with GGG < 3 and GGG ≥ 3 [18] (or GGG < 2 and GGG ≥ 2 [54]) were defined as having non-csPCa and csPCa, respectively. Table 1 [31, 54, 65–84] listed the studies for the diagnosis field of PCa, which were included in the present study.

**Table 1:** Baseline characteristics of studies in the diagnosis field of prostate cancer

No.	Study ID	Years	Country	Diagnosis task	Number of centers	Number of patients	AI methods	Clinical assessment methods	Comparison^#^
1	Hiremath et al. [31]	2021	USA	csPCa	5	592	DL(2D)	Expert reader	>
								Expert reader and clinical characteristics^†^	>
2	Schelb et al. [54]	2019	Germany	csPCa	1	312	DL(2D)	Expert reader	≈
3	Winkel et al. [65]	2020	Switzerland	csPCa	1	201	HC	Expert reader	> (PZ)
4	Dinh et al. [66]	2018	France	csPCa	2	235	HC	Expert reader	≈ (PZ)
								Less-experience	> (PZ)
5	Netzer et al. [67]	2021	Germany	csPCa	2	1488	DL(2D)	Expert reader	≈
6	Zhong et al. [68]	2019	USA	csPCa	1	140	DL(2D)	Expert reader	≈
7	Deniffel et al. [69]	2020	Canada	csPCa	1	499	DL(3D)	Expert reader	>
								Expert reader and PSA density^†^	>
8	Liu et al. [70]	2021	USA	csPCa	1	402	HC, DL(3D)	Expert reader	>
9	Zhao et al. [71]	2023	China	csPCa, PCa	7	1861	DL(3D)	Expert reader	≈
10	Youn et al. [72]	2021	Korea	csPCa	1	121	DL(3D)	Expert reader	<
								Less-experience	≈
								Residents	>
11	Yu et al. [73]	2023	China	csPCa	4	1540	DL(2D)	General radiologist	> (internal)
								General radiologist	≈ (external)
								Expert reader	<
12	Hectors et al. [74]	2021	USA	csPCa	1	240	HC	PSA density^†^	>
								Prostate volume^†^	>
13	Hou et al. [75]	2020	China	csPCa	1	263	HC	Expert reader	>
14	Wang et al. [76]	2017	China	PCa	1	54	HC	Expert reader	> (TZ)
								Expert reader	≈ (PZ)
15	Li et al. [77]	2021	China	PCa	1	203	HC	Expert reader	≈
16	Song et al. [78]	2018	China	PCa	1	195	DL(2D)	Reader	>
17	Aussavavirojekul et al. [79]	2022	Thailand	PCa	1	101	HC	Reader	>
18	Kan et al. [80]	2020	China	PCa	2	346	HC	Expert reader	>
19	Antonelli et al. [81]	2019	UK	Gleason pattern 4	1	164	HC	Expert reader	>
20	Niu et al. [82]	2018	China	High grade	1	184	HC	Reader	>
21	Algohary et al. [83]	2020	USA	D’Amico	4	231	HC	Reader	>
22	Zhang et al. [84]	2019	China	Risk	1	59	HC	Clinical characteristics^†^	>

*AI* artificial intelligence, *csPCa* clinically significant prostate cancer, *DL(2D)* deep learning model based on two-dimensional networks, *DL(3D)* deep learning model based on three-dimensional networks, *HC* hand-crafted, *PCa* prostate cancer, *PZ* peripheral zone, *PSA* prostate specific antigen, *TZ* transition zone

^#^Comparison between AI and clinical assessment methods

^†^Diagnostic models based on clinical characteristics

### Comparing the performances of AI and clinical assessment methods for csPCa diagnosis

**Fig. 2:**
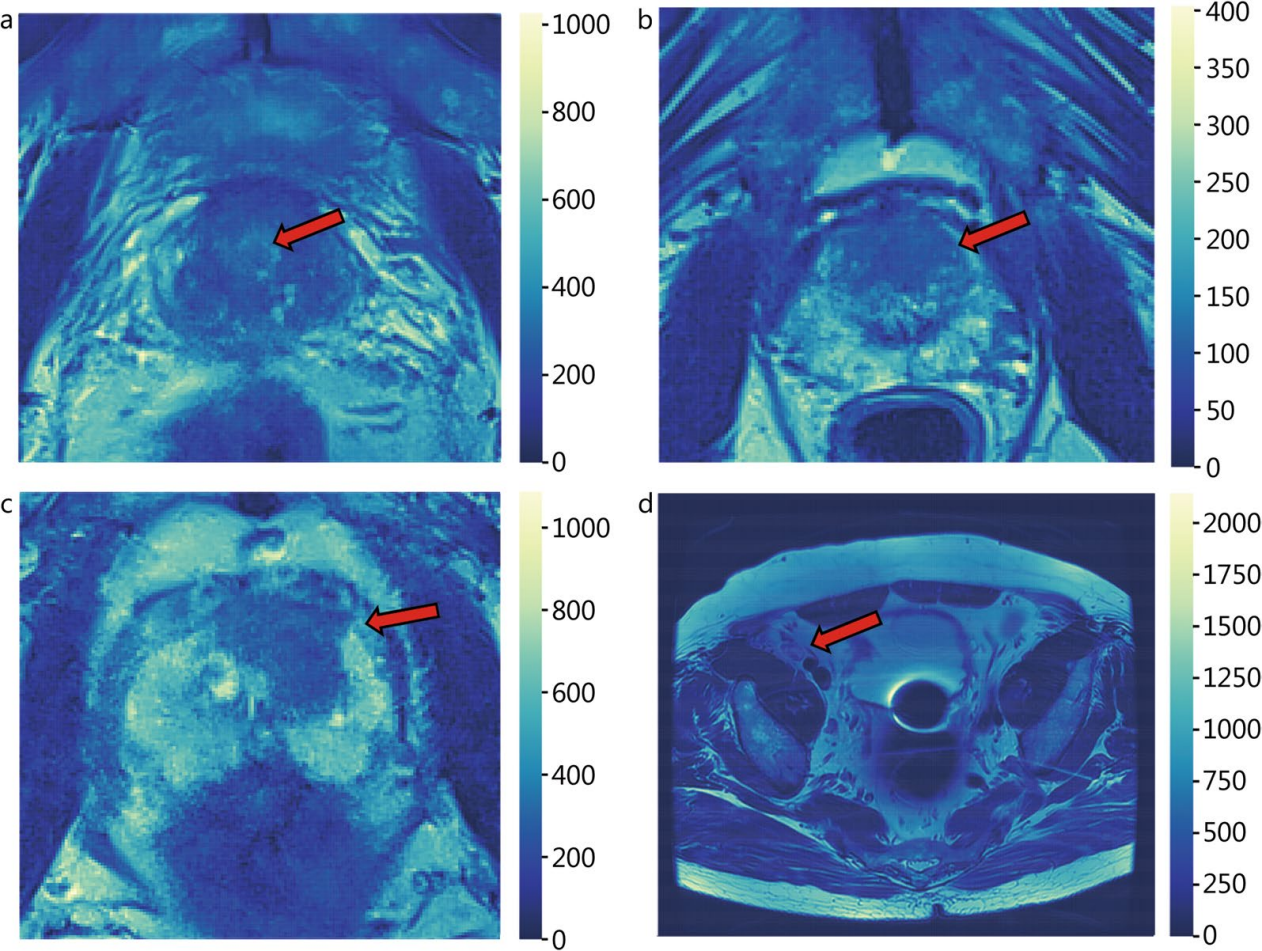
Representative examples of patients with prostate cancer. **a** Patient with non-csPCa; **b** patient with csPCa; **c** patient with ECE; **d** patient with LNI. The colorbar indicates the gray intensity of corresponding voxel in each sub-figure. The hot and cold colors indicate the strong and weak gray intensity, respectively. The red arrow points to the location of the lesion region. csPCa clinically significant prostate cancer, ECE extracapsular extension, LNI lymph node involvement

In clinical practice, patients with a high risk of csPCa assessed using MRI are recommended to undergo biopsies for further pathological confirmation. Therefore, accurate identification of csPCa candidates can help to reduce unnecessary biopsies. Figure 2a and b shows representative examples of patients with non-csPCa and csPCa, respectively. MRI interpretation following the PI-RADS guidelines has been used as a common clinical assessment method for csPCa diagnosis. Thus, most studies have compared the performance of AI methods with that of radiologists’ interpretations of PI-RADS.

Some studies reported that AI models achieved performances similar to or better than those of clinical PI-RADS assessments (Table 1). Winkel et al. [65] proposed several HC models based on quantitative radiomics features. All models outperformed the PI-RADS assessment for the detection of csPCa in the peripheral zone (PZ). Dinh et al. [66] trained a computer-aided diagnosis system to identify csPCa in the PZ based on hand-crafted radiomics features, which achieved comparable performance to those of experienced radiologists and higher performance than those of less-experienced radiologists. Schelb et al. [54] used a DL model [i.e., a two-dimensional (2D) U-Net] trained with bi-parametric MRI (bpMRI) to achieve automatic segmentation and csPCa detection. The U-Net showed good agreement with the PI-RADS assessment by comparing the sensitivities and specificities. Similarly, Netzer et al. [67] confirmed that 2D U-Net could achieve a performance similar to that of PI-RADS assessment for the identification of csPCa. Zhong et al. [68] indicated that a DL model (i.e., a 2D ResNet) that used a transfer-learning method could distinguish indolent from csPCa lesions based on bpMRI, achieving a comparable performance to that of PI-RADS assessment. Deniffel et al. [69] used a DL model [i.e., a shallow 3D convolutional neural network (CNN)] based on bpMRI to diagnose csPCa. They revealed that the model resulted in fewer unnecessary biopsies compared to clinical assessment methods [e.g., PI-RADS-only assessment or the combination of PI-RADS assessment and PSA density (PSAD)]. Hiremath et al. [31] developed an integrated nomogram combining 2D deep learning, PI-RADS, and clinical characteristics, using a logistic regression method to identify csPCa. They found that the nomogram outperformed the PI-RADS assessment and a diagnostic model based on the combination of PI-RADS and clinical characteristics for diagnosing csPCa. Liu et al. [70] combined hand-crafted radiomics features and a deep learning method to diagnose csPCa by integrating a 3D gray-level co-occurrence matrix extractor into a deep learning network. The model was superior to PI-RADS assessment for detecting csPCa. Zhao et al. [71] developed 3D DL models based on multi-center bpMRI for diagnosing csPCa, which showed comparable performance to PI-RADS assessments of expert-level radiologists. Further, the integrated model combining the DL signature and PI-RADS assessment score achieved higher or equal area under the receiver operating characteristic curve (AUC) and greatly increased the specificity compared to PI-RADS assessment in the diagnosis of csPCa.

However, two recent studies reported decreased performance of a DL model for diagnosing csPCa compared to radiologists’ assessments. Specifically, Youn et al. [72] found that, for the diagnosis of csPCa, the AUC of the DL method was similar to that of less-experienced radiologists but lower than that of experts. However, the sensitivity and specificity of the DL model were comparable to those of experts at a threshold PI-RADS score ≥ 4. Yu et al. [73] developed a DL model that segmented prostate lesions automatically and diagnosed caPCa, whose performance was comparable or superior to general radiologists, but inferior to expert-level radiologists in diagnosing csPCa. Additionally, some other studies also developed the two-stage DL models including automatic lesion segmentation and diagnosis, though they did not compare their respective models with clinical assessment [85–87]. These studies suggested that automatic delineation of prostate lesions is of vital importance to reduce the burden on radiologists and improve diagnostic accuracy.

Some recent studies using AI methods focused on the diagnosis of csPCa in lesions with a PI-RADS score 3 because they were equivocal for detecting csPCa [25, 26]. For example, Hectors et al. [74] constructed an HC model to identify csPCa in lesions with a PI-RADS score 3. The model achieved a higher AUC than the diagnostic model based on clinical characteristics (e.g., PSA density or prostate volume). Hou et al. [75] developed an HC model to diagnose csPCa from lesions with a PI-RADS score 3. The model showed better performance than the reassessment results of expert radiologists.

The diagnosis of csPCa is a typical task in the diagnosis field of PCa, accounting for the largest proportion of the included studies (Table 1). For further comparison of the performance between AI and clinical assessment methods in csPCa diagnosis, we calculated the area under the summary receiver operator characteristic curve (SROC-AUC), pooled sensitivity, and pooled specificity of these two methods for csPCa diagnosis among the above-mentioned studies. The pooled sensitivity and specificity of each method (i.e., AI and clinical assessment) were calculated based on the summation of the true positive (TP), false positive (FP), false negative (FN), and true negative (TN) across all included studies about csPCa diagnosis (Table 1). As summarized in Table 2, the AI methods of the studies on csPCa diagnosis presented an SROC-AUC of 0.87, a pooled sensitivity of 0.90, and a pooled specificity of 0.60. In contrast, the clinical assessment methods of all studies of csPCa diagnosis presented an SROC-AUC of 0.82, a pooled sensitivity of 0.93, and a pooled specificity of 0.46. Compared with clinical assessment methods, AI methods achieved higher specificity with slight decrease in sensitivity. Additionally, in terms of SROC-AUC, the AI methods were superior to the clinical assessment methods.

**Table 2:** Comparison of AI and clinical assessment methods in the diagnosis field of PCa

Comparison	AI methods		Clinical assessment methods
	HC model	DL model	
Overall performance	Relatively high		Relatively poor
SROC-AUC^*^	0.87		0.82
Pooled sensitivity^*^	0.90		0.93
Pooled specificity^*^	0.60		0.46
Qualitative or quantitative	Quantitative		Semi-quantitative
Expert dependence	Moderate	Low	High
Consistency	High		Low
Manual delineation	Yes	No	No
Features	High-throughput features extracted using specific algorithms (e.g., shape, histogram, and textural features)	Automatic extraction of deep and subtle image features using networks with substantial parameters	Features for visual assessments (e.g., location, shape, size, and intensity) and some clinical characteristics

*AI* artificial intelligence, *csPCa* clinically significant prostate cancer, *DL* deep learning, *HC* hand-crafted, *PCa* prostate cancer, *PI-RADS* prostate imaging reporting and data system, *SROC-AUC* area under the summary receiver operating characteristic curves

^*^Performance indexes pooled across the studies on csPCa diagnoses

In general, the total performance of AI methods was better than that of clinical assessments for the studies on csPCa diagnosis. Specifically, for the studies on csPCa diagnosis, AI methods showed higher specificity but slightly less sensitivity compared to clinical assessment methods. In these studies, the clinical assessment methods showed a ceiling-approximate pooled sensitivity but a very low pooled specificity. Thus, the advantages of AI methods are mostly observed in the improved specificity of csPCa diagnosis. Notably, in both studies and clinical practice, PI-RADS is a common guideline for csPCa diagnosis based on medical images [26], which has been reported to have a low specificity [6]. Thus, the comparison results of these studies revealed that AI methods could improve the specificity of csPCa diagnosis, thereby reducing unnecessary confirmatory biopsies.

### Comparing the performances of AI and clinical assessment methods for PCa diagnosis

In clinical practice, misidentifying benign as malignant lesions in patients will cause anxiety and unnecessary trauma. Thus, accurately discriminating between benign (e.g., prostatic hyperplasia and inflammation) and malignant prostate lesions is crucial. MRI interpretation following the PI-RADS guidelines has been used as a common clinical assessment method for PCa diagnosis [25, 26]. Thus, most studies have compared the performance between AI methods and radiologists’ interpretation of PI-RADS.

Several studies have reported that AI models can aid radiologists in PCa diagnosis by improving their visual assessments (Table 1). Wang et al. [76] reported that an HC model could improve the performance of PI-RADS assessment in diagnosing PCa, especially for lesions in the transitional zone (TZ). Li et al. [77] found that the difference of AUC between HC model and PI-RADS was insignificant. However, the integration of HC model and PI-RADS better diagnosed PCa than the PI-RADS assessment. Song et al. [78] reported that a joint model of DL and PI-RADS assessment outperformed either the DL model or PI-RADS assessment in diagnosing PCa. Zhao et al. [71] developed 3D DL models based on multi-center bpMRI for diagnosing PCa, which showed comparable performance to PI-RADS assessments of expert-level radiologists. Additionally, both Aussavavirojekul et al. [79] and Kan et al. [80] constructed HC models to detect PCa from lesions with a PI-RADS score 3, achieving specificities of 72% and 50%, respectively. It should be noted that in these two studies (i.e., [79] and [80]), equivocal lesions with PI-RADS score 3 were confirmed by biopsies. Thus, the AI methods in these studies can aid decision-making regarding whether a lesion with a PI-RADS score 3 should undergo biopsy confirmation. Further, Luo et al. [88] reported that an AI-based image reconstruction algorithm might increase the MRI resolution and thereby improve the display effect, aiding in a better identification of PCa from benign prostatic hyperplasia.

### Comparing the performances of AI and clinical assessment methods for other diagnostic tasks for risk stratification of PCa

In addition to diagnosing PCa and csPCa, AI methods have been used to aid other diagnostic tasks for risk stratification of PCa. Several studies have reported that AI models can aid radiologists in improving their visual assessments of other diagnostic tasks (Table 1). For example, Antonelli et al. [81] found that an HC method showed better performance in recognizing lesions with Gleason pattern 4 than three board-certified radiologists’ assessments. Niu et al. [82] reported that an HC model could detect high-grade PCa and performs better than PI-RADS assessment. Algohary et al. [83] developed an HC model combining peritumoral and intratumoral radiomics features to accurately stratify PCa risk that was defined by the D’Amico Risk Classification System, resulting in higher accuracy compared to the PI-RADS assessment. Zhang et al. [84] used the logistic regression method combining hand-crafted radiomics features and clinical characteristics to differentiate between high- and low-grade PCa, which outperformed the diagnosis model based on clinical characteristics.

### Summary of the comparison between AI and clinical assessment methods in the diagnosis field of PCa

Table 2 summarized the differences between AI and clinical assessment methods for the diagnosis field of PCa. First, AI methods achieved a better overall performance than that of clinical assessments of radiologists for the diagnosis of PCa. In particular, for the detection of csPCa, the SROC-AUC and pooled specificity of AI methods were both higher than those of the clinical assessment methods. Further, different from the clinical assessment methods, the AI methods provided a quantitative result, which relied much less on the individual expertise of radiologists and thereby could achieve consistent diagnosis results. The better performance of AI compared with clinical assessment methods was because the former can mine subtle and deep information of MR images of PCa, which was not accessible by common clinical assessment methods. Though the HC methods required precisely manual delineations of prostate lesions, the DL methods achieved an automatic image-to-decision diagnosis.

**Fig. 3:**
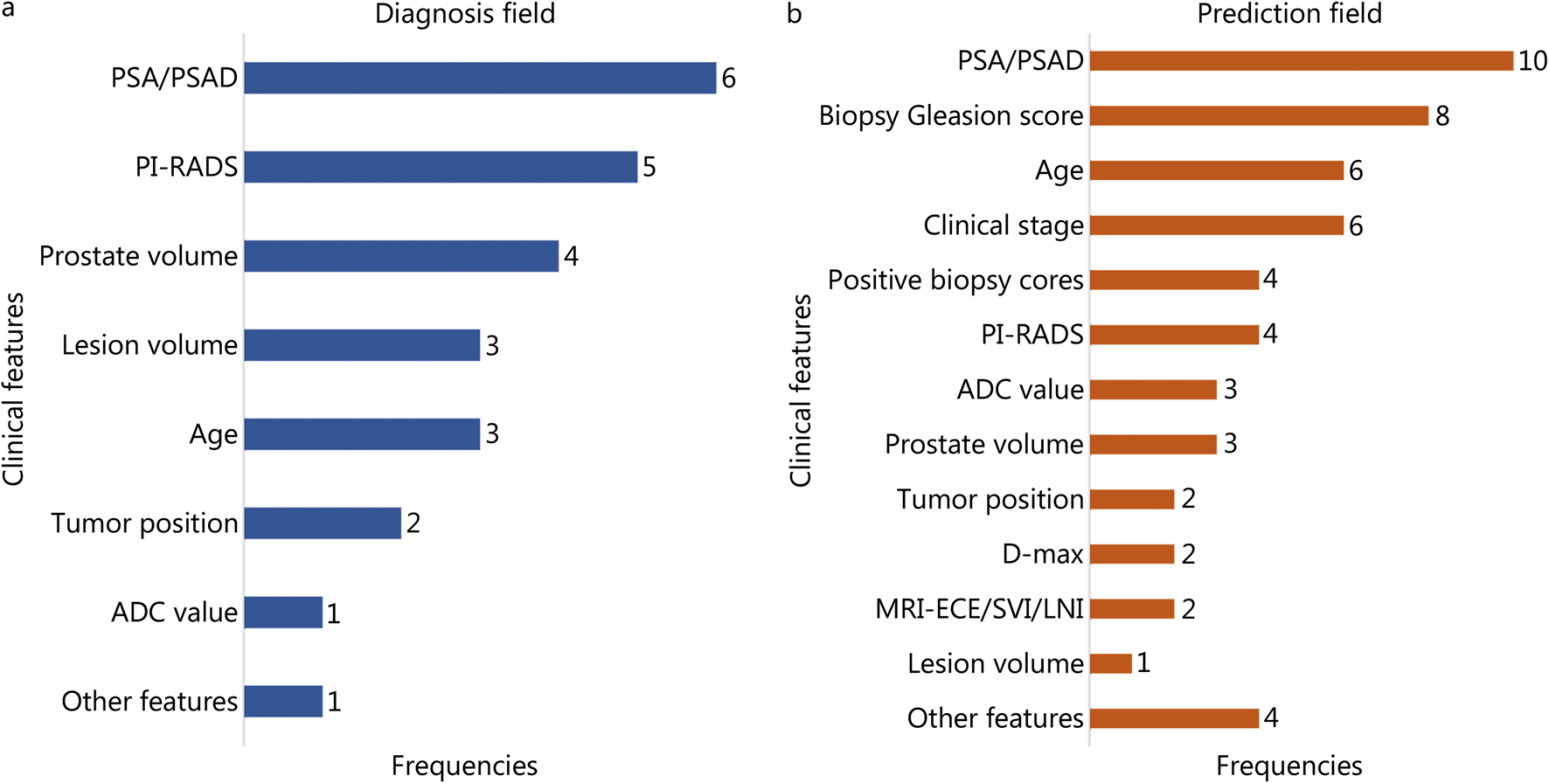
Frequencies with which the clinical features were combined with AI models based on MRI in the diagnosis (**a**) and prediction (**b**) field of PCa. ADC apparent diffusion coefficient, AI artificial intelligence, D-max the lesion maximum cross-sectional diameter, MR magnetic resonance, MRI-ECE MRI-based extracapsular extension, MRI-LNI MRI-based lymph node involvement, MRI-SVI MRI-based seminal vesicle invasion, PCa prostate cancer, PI-RADS prostate imaging reporting and data system, PSA prostate specific antigen, PSAD prostate specific antigen density

Because the image features and clinical features include different information for characterizing prostate lesions, the combination of the clinical characteristics and the AI models based on MR images improves the performance of diagnosing PCa (e.g., [31, 71]). As shown in Fig. 3a, for the integrated AI models included in the present study, the clinical characteristics of PSA/PSAD, PI-RADS, and prostate volume were frequently combined with the AI models.

Additionally, in some of the included studies, AI models were trained and tested using a large number of samples from two- or multi-centers [31, 66, 67, 71, 73, 80, 83], demonstrating, to some degree, the potential robustness and generalization of these models in the diagnosis field of PCa. These findings suggest that AI models may effectively aid radiologists in improving the diagnosis field of PCa. Furthermore, as in most studies, PI-RADS is a common clinical assessment method for the diagnosis field of PCa in clinical practice. However, as a semi-quantitative scoring system, PI-RADS is associated with low specificity in the diagnosis field of PCa [6], leading to unnecessary confirmation using biopsies [18]. Thus, the combination of AI models and PI-RADS assessment potentially reduces over-biopsies by improving specificity in the diagnosis field of PCa.

However, these studies had several limitations regarding the AI-assisted diagnosis of PCa. First, although two- or multi-center samples were used in a few studies, most employed small and monocenter patient cohorts. Thus, the generalizability of the proposed AI models for the diagnosis field of PCa requires further validation. Second, most deep learning radiomics models for PCa diagnosis were constructed based on a 2D CNN. However, for models with 2D CNN, the final patient-level output results are usually the average of the predicted values of multiple slice images without considering the spatial relationship between slices [89]. Three-dimensional (3D) CNN can fully utilize spatial information and achieve accurate patient-level prediction. Thus, 3D deep-learning radiomics models should be used in future research. Third, some studies used independent external validation cohorts to evaluate model performance; however, they were generally retrospective. Therefore, these models should be validated using prospective data. Finally, HC models still require precise manual annotation, which is time-consuming and laborious, and requires automatic annotation for PCa lesions.

In general, when applied to the analysis of medical images, AI methods present advantages over clinical assessments in various aspects, such as the high-throughput extraction of image information, overall characterization of lesion heterogeneity, and multi-variable analysis of image features. Thus, it was not unexpected that the AI methods outperformed the clinical assessment methods in the diagnosis field of PCa. The advantages of AI methods over clinical assessment methods are highly consistent with studies on other tumors, such as breast cancer [90, 91], brain tumor [92], renal cancer [93], and cervical cancer [94], suggesting that AI methods are potential tools to aid in the precise diagnosis of PCa.

## Comparing the performances of AI and clinical assessment methods in the prediction field of PCa

AI methodology has been widely utilized to aid in the prediction fields of PCa. In clinical practice, the prediction tasks mainly include predicting lymph node involvement (LNI), ECE, postoperative BCR, and other events. Table 3 [57, 58, 95–108] listed the studies for the prediction field of PCa, which were included in the present study.

**Table 3:** Baseline characteristics of studies in the prediction field of prostate cancer

No.	Study ID	Years	Country	Prediction task	Number of centers	Number of patients	AI methods	Clinical assessment methods	Comparison^#^
1	Yan et al. [57]	2021	China	BCR	3	485	HC, DL(2D)	CAPRA-S/NCCN/GGG	>
2	Hou et al. [58]	2021	China	ECE	2	849	DL(2D)	Expert reader	>
3	Xu et al. [95]	2020	China	ECE	1	95	HC	Clinical and pathological characteristics^†^	>
4	Ma et al. [96]	2019	China	ECE	1	210	HC	Reader	>
5	Bai et al. [97]	2021	China	ECE	2	284	HC	Clinical characteristics^†^	< (internal) ≈ (external)
6	Bourbonne et al. [98]	2021	France	LNI	1	280	HC, DL(2D)	Partin/Roach/Yale/MSKCC/…	>
7	Hou et al. [99]	2019	China	LNI	1	248	HC	MSKCC	>
8	Hou et al. [100]	2021	China	LNI	2	401	HC, DL(2D)	MSKCC/Briganti/…	>
9	Li et al. [101]	2021	USA	AP, BCR	4	198	HC	CAPRA/Decipher/CAPRA-S	>/≈
10	Bourbonne et al. [102]	2020	France	BCR	2	195	HC	Clinical characteristics^†^	>
11	Sushentsev et al. [103]	2022	UK	Progressing	1	64	HC	PRECISE	≈
12	Xie et al. [104]	2021	China	Upgrade	1	59	HC	Biopsy	>
13	Zhang et al.[105]	2020	China	Upgrade	1	166	HC	Clinical characteristics^†^	>
14	Wu et al. [106]	2017	China	Upgrade	1	46	HC	Clinical characteristics^†^	>
15	Zheng et al. [107]	2021	USA	Biopsy results	1	330	HC	PSA density^†^	>
16	Wang et al. [108]	2018	China	Organ-confined Pca	1	541	HC	Partin	>

*AI* artificial intelligence, *AP* adverse pathology, *BCR* biochemical recurrence, *CAPRA* Cancer of the Prostate Risk Assessment, *DL(2D)* deep learning model based on two-dimensional networks, *ECE* extracapsular extension, *GGG* Gleason grade group, *HC* hand-crafted, *LNI* lymph node involvement, *MSKCC* Memorial Sloan Kettering Cancer Center, *NCCN* National Comprehensive Cancer Network, *PSA* prostate specific antigen, *PRECISE* Prostate Cancer Radiological Estimation of Change in Sequential Evaluation

^#^Comparison between AI methods and clinical assessment methods

^†^Predictive models based on clinical characteristics

### Comparing the performances of AI and clinical assessment methods for AP prediction

AP features (e.g., ECE and LNI) are known to be important predictors of tumor metastasis, and therefore, accurate prediction of the presence of AP features can significantly aid in treatment decisions (e.g., planning of personalized surgical treatment) [10, 15]. Figure 2c and d show representative examples of patients with ECE and LNI, respectively. In clinical practice, radiologists’ interpretations and nomograms based on clinical characteristics (e.g., PSA level, Gleason grade, and positive biopsy cores) are commonly used as clinical assessment methods to predict AP. Thus, most studies have compared the performance between AI methods and radiologists’ interpretations or nomograms based on clinical characteristics.

Several studies have reported that the performance of AI models is equal to or better than that of clinical assessment methods (Table 3). Hou et al. [58] developed a DL model that contained an attention map of experts’ prior knowledge to detect ECE. It showed better performance than radiologists’ interpretations. The study also reported that the performance of radiologists’ interpretations in ECE prediction was improved with the assistance of the DL model. Xu et al. [95] built an HC model to predict ECE that outperformed a prediction model combining clinical and pathological characteristics. Ma et al. [96] also constructed an HC model to predict the presence of ECE that outperformed radiologists’ interpretations. Additionally, some studies have reported that prediction models combining radiomics features and clinical characteristics achieved excellent performance. For example, Bai et al. [97] constructed a logistic regression model combining peritumoral hand-crafted radiomics features and clinical characteristics to predict ECE. The model achieved comparable or better performance than a prediction model based on clinical characteristics. Bourbonne et al. [98] proposed a DL model combining hand-crafted radiomics features and clinical characteristics to predict LNI in PCa patients. The model provided a higher C-index than other clinical nomograms [i.e., Partin, Roach, Yale, and Memorial Sloan Kettering Cancer Center (MSKCC)]. Hou et al. [99] selected 18 features, including hand-crafted radiomics features and clinical characteristics, and developed several models (i.e., logistic regression, SVM, and RF) to predict LNI. The predictive performances of these models were superior to those of the MSKCC nomogram. Hou et al. [100] used an RF model combining clinicopathological factors, radiologists’ interpretations, hand-crafted radiomics features, and deep learning radiomics features to predict LNI. The performance of the model was superior to those of the MSKCC, Briganti and any other model based on a single type of characteristic or a combination of the two types of characteristics in the internal and external testing cohorts. Li et al. [101] developed a nomogram combining hand-crafted radiomics and clinicopathologic features to predict the presence of AP of PCa. The nomogram outperformed the Cancer of the Prostate Risk Assessment (CAPRA) and the Decipher test for predicting the presence of AP.

AP prediction is a common task in the prediction field of PCa accounting for the largest proportion of the included studies (Table 3). For further comparison of the performance between AI and clinical assessment methods in AP prediction, we calculated the SROC-AUC, pooled sensitivity, and pooled specificity of these two methods among the above-mentioned studies. As summarized in Table 4, the AI methods for AP prediction presented an SROC-AUC of 0.86, a pooled sensitivity of 0.75, and a pooled specificity of 0.84. In contrast, the clinical assessment methods of AP feature prediction presented an SROC-AUC of 0.75, a pooled sensitivity of 0.68, and a pooled specificity of 0.79. In terms of the above performance indexes, AI methods were superior to clinical assessment methods.

**Table 4:** Comparison of AI and clinical assessment methods in the prediction field of PCa

Comparison	AI methods		Clinical assessment methods
	HC model	DL model	
Overall performance	Relatively high		Relatively poor
SROC-AUC^*^	0.86		0.75
Pooled sensitivity^*^	0.75		0.68
Pooled specificity^*^	0.84		0.79
Qualitative or quantitative	Quantitative		Quantitative/qualitative
Expert dependence	Moderate	Low	High
Consistency	High		Moderate
Manual delineation	Yes	No	No
Features	High-throughput features extracted using specific algorithms (e.g., shape, histogram and textural features)	Automatic extraction of deep and subtle image features using networks with substantial parameters	Clinical characteristics (e.g., PSA, Gleason grade and positive biopsy cores) or features for visual assessments (e.g., location, shape, size, and intensity)

*AI* artificial intelligence, *AP* adverse pathology, *DL* deep learning, *HC* hand-crafted, *PCa* prostate cancer, *PSA* prostate specific antigen, *SROC-AUC* area under the summary receiver operator characteristic curves

^*^Performance indexes pooled across the studies of AP prediction

### Comparing the performances of AI and clinical assessment methods for BCR prediction

In clinical practice, patients with BCR after RP or other therapies may present more advanced disease, distant metastasis, and even death [52]. Thus, early identification of BCR can help to make treatment decisions.

Recently, AI has been widely used for BCR prediction [57, 101, 102, 109, 110]. Several studies have compared AI and clinical assessment methods and demonstrated that the performance of AI models is better than that of clinical assessment methods for BCR prediction (Table 3). For example, Yan et al. [57] extracted hand-crafted radiomics features and developed a DL model to predict BCR after RP with MR images, which outperformed other clinical assessment methods (e.g., CAPRA-S score, NCCN model, and Gleason grade group systems). Li et al. [101] developed a nomogram combining hand-crafted radiomics features and clinicopathologic features to predict the post-surgical BCR of PCa. The nomogram yielded a higher C-index than CAPRA and Decipher and was equal to CAPRA-S for the prediction of BCR. Bourbonne et al. [102] trained an HC model to predict the BCR for high-risk PCa, which outperformed other prediction models based on clinical characteristics.

### Comparing the performances of AI and clinical assessment methods for other predictive tasks for PCa prognosis

In addition to predicting AP and BCR, AI methods have been used to aid other predictive tasks for PCa. Several studies have reported that the performance of AI models is equal to or better than that of clinical assessment methods for other predictive tasks (Table 3). For example, Sushentsev et al. [103] developed an HC model for predicting PCa progression in patients undergoing active surveillance. The model’s performance was comparable to that of the clinical assessment method [i.e., Prostate Cancer Radiological Estimation of Change in Sequential Evaluation (PRECISE)]. Xie et al. [104] extracted textural features from ADC maps and developed HC models to predict pathological upgrading from biopsy to RP. These models showed excellent performance, suggesting that they can improve the diagnostic accuracy of biopsy and avoid missed detection of high-grade PCa. Zhang et al. [105] built a logistic regression model combining hand-crafted radiomics features and clinical characteristics to predict upgrading from biopsy to RP. It outperformed the prediction model based on clinical characteristics including clinical stage and time from biopsy to RP. Wu et al. [106] developed a logistic regression model combining diffusion kurtosis imaging and PSA to predict upgrading after RP. It outperformed other prediction models based on clinical characteristics. Zheng et al. [107] trained an SVM model combining hand-crafted radiomics features and clinical characteristics to predict biopsy results for patients with negative MRI findings. The proposed model was superior to PSA density-based risk assessment. Wang et al. [108] developed an SVM model combining clinical characteristics (i.e., age, PSA, clinical stage, and biopsy Gleason score) and MRI findings (i.e., tumor location, PI-RADS scores, diameter, and 6-point MRI stage) for the prediction of organ-confined PCa, which outperformed the clinical assessment method (i.e., Partin table).

### Summary of the comparisons between AI and clinical assessment methods in the prediction field of PCa

Table 4 summarized the differences between AI and clinical assessment methods for the prediction field of PCa. First, AI methods achieved a better overall performance than that of clinical assessments of radiologists for the prediction of PCa. In particular, for the prediction of AP presence, all the SROC-AUC, pooled sensitivity and pooled specificity of AI methods were higher than those of the clinical assessment methods. Although both AI and clinical assessment methods can provide a quantitative result, the former rely much less on the individual expertise of radiologists and thereby could achieve consistent prediction results. Additionally, the AI methods can extract high-throughput features and subtle information, the majority of which were not accessible by the clinical assessment methods.

Like those in the diagnosis field of PCa, the integrated AI models combing the AI models based on MR images and clinical characteristics achieved an increased performance for the prediction of PCa (e.g., [100, 101]). As shown in Fig. 3b, for the integrated AI models included in the prediction field, the clinical characteristics of PSA/PSAD, biopsy Gleason score, age, clinical stage (C-stage), positive biopsy cores, and PI-RADS were frequently combined with the AI models.

Overall, studies in the prediction field of PCa mostly focused on the prediction of LNI, ECE, BCR, and pathological upgrading from biopsy to RP. All above-mentioned studies reported better or comparable performances of AI models compared to those of clinical assessment methods. In particular, in some of these studies, the AI models were tested using an external testing cohort [57, 58, 97, 100–102], demonstrating, to some degree, the potential robustness and generalization of these models in clinical application to the prediction field of PCa. These findings suggest that AI models may effectively improve preoperative prediction performance and assist clinicians in making treatment decisions. Furthermore, as in some studies, some risk assessment tools, such as Partin tables, MSKCC nomogram, and CAPRA score, showed moderate predictive performance on validation [57, 98, 100, 101]. Additionally, most of studies employed HC models as prediction methods, the number of which was much larger than that of studies using DL models. Furthermore, all studies using DL models employed 2D CNN without considering the 3D spatial information of tumors.

In clinical practice, radiologists’ interpretations and nomograms, such as CAPRA scores and MSKCC nomograms, are commonly used to predict therapeutic outcomes. These nomograms integrated multiple clinicopathological features, such as PSA, Gleason grade, and positive biopsy cores, but failed to account for tumor heterogeneity, resulting in relatively poor performance. MRI can visually and comprehensively describe the characteristics and morphology of tumors associated with tumor aggressiveness and progression [15]. However, MRI interpretation based on some evidently visual features of lesions (e.g., size, location, and intensity) requires a high level of expertise by radiologists, leading to interobserver variability. Furthermore, lesions with low volumes may be missed in visual assessments, and various invisible features (e.g., subtly textural and advanced features) have also been associated with PCa aggressiveness and progression. In contrast, AI methods can automatically extract features from images, reducing the dependence on the high-level expertise of radiologists. Additionally, AI methods can extract visible features and mine invisible high-throughput information, thus overcoming the limitations of radiologists’ interpretations. The advantages of AI methods over clinical assessment methods were highly consistent with those of studies on other tumors, such as breast cancer [36, 90], brain tumor [92, 111, 112], rectal cancer [37, 113, 114], gastric cancer [41, 114, 115], colon cancer [116], lung cancer [117–119], and cervical cancer [120, 121], suggesting that AI methods are potential tools to aid the precise prediction of PCa.

## RQS assessment

**Fig. 4:**
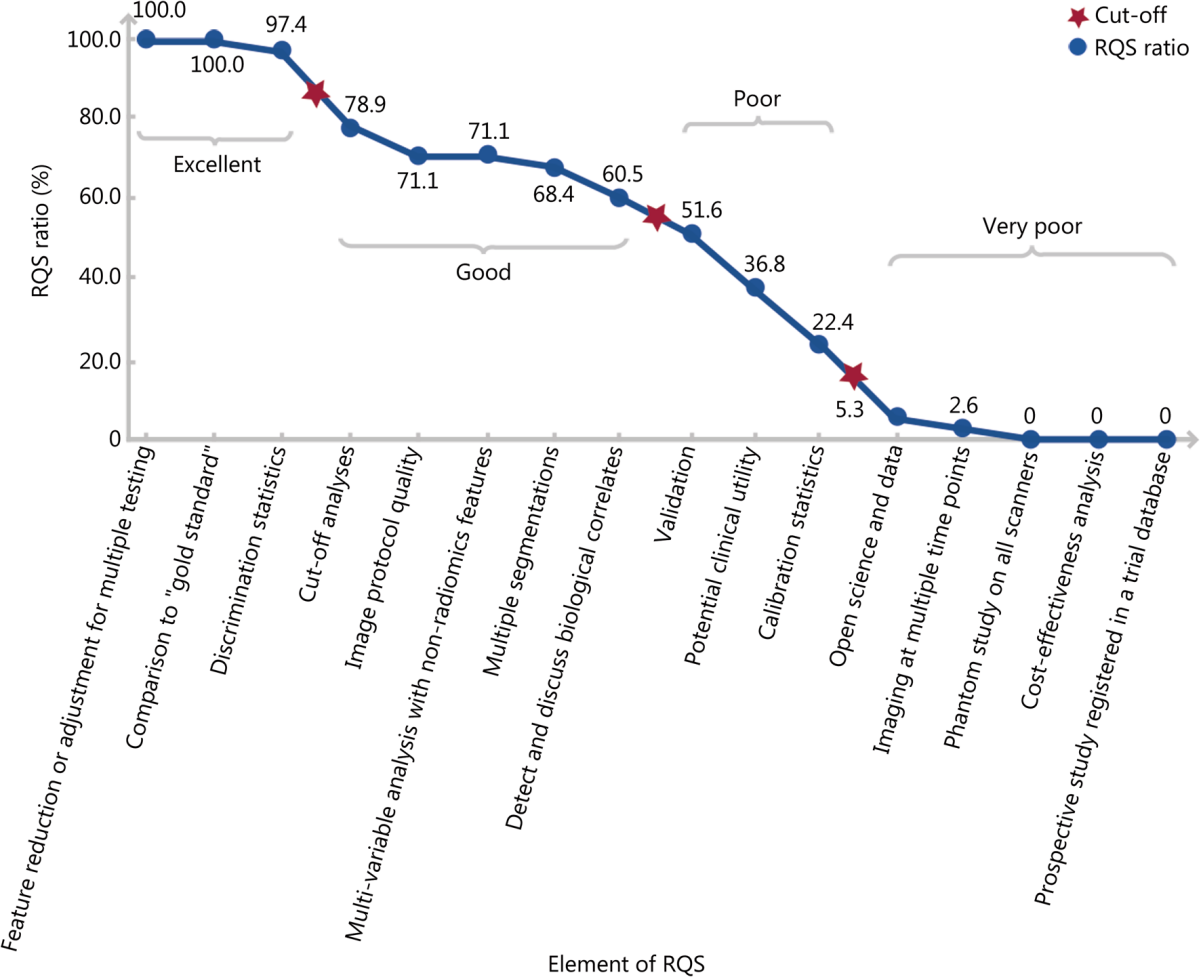
RQS results of AI models for all included studies in diagnosis and prediction fields. AI artificial intelligence, RQS radiomics quality score

The RQS assessment [122] was performed for all included studies in both diagnosis (Table 1) and prediction (Table 3) fields of PCa of the present study. The RQS assessments were conducted independently by two reviewers. The disagreement between the reviewers was resolved by discussion until achieving an agreement. These studies presented a total average RQS of 15.2 (11.0–20.0) with a total average RQS ratio of 42.2%, which was defined as the ratio of the total average RQS to the full points (i.e., 15.2/36). This total average RQS ratio is higher than those of some recent radiomics studies [123–125] (average RQS ratio: 14.3–29.6%). The RQS ratio of each RQS element was defined as the ratio of the average RQS across all included studies to the full points for the corresponding element, which reflected the degree to which all included studies met the requirements of the corresponding RQS element. When the RQS elements were sorted in descending order of their RQS ratios, they were divided into four levels by three evident cutoffs: excellent, good, poor, and very poor (Fig. 4).

As shown in Fig. 4, the excellent elements (i.e., RQS ratio = 97.4–100.0%) included “feature reduction or adjustment for multiple testing”, “comparison to gold standard”, and “discrimination statistics”. The good elements (i.e., RQS ratio = 60.5–78.9%) included “cut-off analyses”, “image protocol quality”, “multi-variable analysis with non-radiomics features”, “multiple segmentations”, and “detect and discuss biological correlates”. Some of the excellent and good elements (e.g., “feature reduction or adjustment for multiple testing”, “comparison to gold standard”, “discrimination statistics”, “cut-off analyses”, and “multiple segmentations”) are closely related to several processes of AI model construction, such as the extraction and selection of radiomics features, as well as the assessment and comparison of model performance, suggesting the relatively perfect and standard construction processes of AI models for the included studies. Additionally, the RQS elements of “detect and discuss biological correlates” and “multi-variable analysis with non-radiomics features” also presented a relatively high RQS ratio, highlighting some clinical significance of the included studies.

Poor elements (i.e., RQS ratio = 22.4–51.6%) included “validation”, “potential clinical utility”, and “calibration statistics”. The very poor elements (i.e., RQS ratio = 0–5.3%) included “open science and data”, “imaging at multiple time points”, “phantom study on all scanners”, “cost-effectiveness analysis”, and “prospective study registered in a trial database”. It is noted that, among these elements, three have the top three full points, namely “prospective study registered in a trial database” (full points of 7), “validation” (full points of 5), and “open science and data” (full points of 4). However, they presented relatively low RQS, particularly the element of “prospective study registered in a trial database” even presenting a zero average RQS ratio, suggesting that none of the included studies used prospective data to test the AI models. These three RQS elements are related to the assessment of the generalizability and replicability of the AI models. Therefore, these low RQS ratios suggest that the robustness of AI models in most of the included studies is unclear. Additionally, the elements of “phantom study on all scanners”, “imaging at multiple time points”, and “cost-effectiveness analysis” also had very low average RQS ratios. This may be because all included studies were retrospective. Considering that “phantom study on all scanners” and “imaging at multiple time points” facilitates the examination of feature robustness to inter-scanner differences and temporal variabilities [122], this should be considered in future prospective studies.

According to above description, the RQS element can be mostly categorised into two groups. One is related to the performance improvement of AI models, such as “feature reduction or adjustment for multiple testing”, “multi-variable analysis with non-radiomics features”, “multiple segmentations” and “phantom study on all scanners”. Specifically, for the element of “feature reduction or adjustment for multiple testing”, feature selection or dimensionality reduction for the extracted features with high redundancy and/or strong collinearity can optimize feature space, thereby improving the performance of the model [74–76, 97, 99]. For the element of “multi-variable analysis with non-radiomics features”, AI can mine subtle information that may reflect heterogeneities of the lesions, but the commonly used clinical characteristics (e.g., PSA, age, family history, and routine habits) also contain information relevant to the diagnosis and prognosis. Thus the clinical characteristics were complementary to the radiomics features of MR images to improve the performance of model [31, 71, 101]. For the element of “multiple segmentations”, the delineation of lesions with different methods (e.g., automatic and manual), by different radiologists and software, and in different stages of the breathing cycles is helpful to reduce the discrepancy between the delineated region of interest (ROI) and the actual lesion. This may heighten the robustness and accuracy of the extracted features, based on which an AI model is constructed [31, 54, 71]. For the element of “phantom study on all scanners”, when radiomics features come from images scanned by multiple scanners, it is important to consider feature variabilities between scanners. The phantom study is an appropriate way to measure the uncertainties of different scanners.

The other group of elements is related to the performance evaluation of the AI model, such as “validation”, “prospective study registered in a trial database”, “cut-off analyses” and “potential clinical utility”. Specifically, for “validation” and “prospective study registered in a trial database”, the testing of an AI model using independent external cohort, particularly the prospective samples, can fully evaluate the robustness and generalisation of the model, thereby reducing the overfitting of AI models. For the element of “cut-off analyses”, some performance indexes, such as sensitivity and specificity, are calculated dependently on the risk threshold. The traditional default value of 0.5 doesn’t exactly reflect the clinical problem. Therefore, the appropriate risk threshold should be selected in conjunction with clinical decisions [54, 67, 71]. For the element of “potential clinical utility”, analyzing potential applications of the model in a clinical practice is of vital importance to make clinical promotion [31, 67, 75].

In addition to the RQS elements, several factors may affect AI performance, such as the development of new AI models, large data queues and interaction between AI methods and clinical problems. First, new breakthroughs of AI technology can provide new networks model with a powerful ability to extract the deep features of medical imaging and combine multiple imaging modalities and multiple time points. This can make the model more fully and accurately characterize the tumor heterogeneity. Second, an AI model trained using a larger data queue based on different institutions and different regions (e.g., cities or countries) can show stronger robustness and generalization. Finally, the development of an AI model that is orientated to a specific clinical problem may make the model have more clinical applicability. Like the RQS elements, these three factors can also effectively improve the performance of AI modes for aiding in the precise diagnoses and treatments of prostate tumors.

Although many proposed AI models have been demonstrated better performance than clinical assessment methods for the diagnosis and prediction of PCa, they are not yet extensively used in the clinical practice. The causes are various and difficult to clearly list. One possible cause may be that the AI models were usually trained using a relatively limited number of samples. Compared to the expertise of clinicians that is accumulated based on tens of years of experience, the limited-trained AI models provide the clinicians with less confidence in the management of PCa. Additionally, the weak interpretability of the AI model may be another cause. Thus, it is very difficult for the clinicians to combine the predicting results of the model with their expertise for the diagnoses and treatments of PCa.

## Conclusions and prospect

In this review, we summarized the studies including the performance comparisons between AI and clinical assessment methods applied to PCa. Several findings were obtained: First, the performance of AI methods was generally better than clinical assessment methods for the diagnosis and prediction fields of PCa, particularly for the detection of csPCa and prediction of some AP features, indicating that AI can aid clinicians in making accurate decisions (e.g., reducing the frequency of unnecessary biopsies and making personalized treatment plans). Second, the AI models were constructed with relatively perfect and standard radiomics processes. However, due to inadequate multi-center validation, prospective data testing, or the opening of the research material, the generalizability and clinical practicality of AI models should be further validated.

In the future, AI models can be improved in the following potential aspects. First, the AI models will be validated using a high level of evidence, such as different race data and prospective data. Second, the combination of radiomics models based on MR images and Natural Language Processing based on medical records will provide more comprehensive information and reduce the burden on radiologists. Third, more state-of-the-art and complex AI methods, such as ones integrating the expertise of radiologists into the latest network architectures, will be developed to further improve the diagnosis and prediction of PCa. Additionally, for the applications of AI methods in the management of PCa, AI methodology should further extend to the fields beyond the above-mentioned tasks in the present study, such as the identification of the patients qualified for active surveillance, the prediction of local recurrence, survival analysis, and comparison of the prognosis of different treatment plans.

## Data Availability

Not applicable.

## Abbreviations

ADC: Apparent diffusion coefficient
AI: Artificial intelligence
AP: Adverse pathology
AUC: Area under the receiver operating characteristic curve
BCR: Biochemical recurrence
bpMRI: Bi-parametric MRI
CAPRA: Cancer of the Prostate Risk Assessment
CNN: Convolutional neural network
csPCa: Clinically significant prostate cancer
CT: Computed tomography
DL: Deep learning
DL(2D): Deep learning model based on two-dimensional networks
DL(3D): Deep learning model based on three-dimensional networks
DWI: Diffusion-weighted imaging
D-max: The lesion maximum cross-sectional diameter
ECE: Extracapsular extension
GGG: Gleason grade group
HC: Hand-crafted
LASSO: Least absolute shrinkage and selection operator
LNI: Lymph node involvement
LR: Logistic regression
MRI: Magnetic resonance imaging
MRI-ECE: MRI-based extracapsular extension
MRI-LNI: MRI-based lymph node involvement
MRI-SVI: MRI-based seminal vesicle invasion
MSKCC: Memorial Sloan Kettering Cancer Center
NCCN: National Comprehensive Cancer Network
PCa: Prostate cancer
PI-RADS: Prostate imaging reporting and data system
PRECISE: Prostate Cancer Radiological Estimation of Change in Sequential Evaluation
PSA: Prostate specific antigen
PSAD: Prostate specific antigen density
PZ: Peripheral zone
RFE: Recursive feature elimination
RP: Radical prostatectomy
RQS: Radiomics quality score
SROC: Summary receiver operator characteristic curve
SROC-AUC: Area under the summary receiver operating characteristic curve
SVM: Support vector machine
T2WI: T2-weighted imaging
TZ: Transition zone
